# Even at 100+: Acute Exercise Modulates Inflammatory Pathways in Centenarians

**DOI:** 10.1111/acel.70152

**Published:** 2025-07-15

**Authors:** Abel Plaza‐Florido, Lidia B. Alejo, Inmaculada Pérez‐Prieto, Pedro Carrera‐Bastos, María Rosado Muñoz, Itziar Pagola Aldazabal, David Barranco‐Gil, Alejandro Santos‐Lozano, Gabriel Rodríguez‐Romo, Natalia Yanguas‐Casás, Shlomit Radom‐Aizik, Alejandro Lucia, Carmen Fiuza‐Luces

**Affiliations:** ^1^ Research Center for Exercise Medicine and Sleep/Pediatric Exercise and Genomics Research Center, Department of Pediatrics, School of Medicine University of California Irvine Irvine California USA; ^2^ Faculty of Medicine, Health and Sports. Department of Sport Sciences Universidad Europea de Madrid Madrid Spain; ^3^ Research Institute of the Hospital 12 de Octubre (‘imas12’) Madrid Spain; ^4^ Department of Obstetrics and Gynecology Copenhagen University Hospital Hvidovre Hvidovre Denmark; ^5^ Universidad Europea de Madrid Faculty of Biomedical and Health Sciences Madrid Spain; ^6^ Center for Primary Health Care Research, Department of Clinical Sciences Lund University Malmö Sweden; ^7^ Rio Maior School of Sport–Santarém Polytechnic University Rio Maior Portugal; ^8^ i+HeALTH Strategic Research Group, Department of Health Sciences Miguel de Cervantes European University Valladolid Spain; ^9^ Deporte y Entrenamiento Research Group, Departamento de Deportes, Faculty of Physical Activity and Sport Sciences (INEF) Universidad Politécnica de Madrid Madrid Spain; ^10^ Centro de Investigación Biomédica en Red de Fragilidad y Envejecimiento Saludable (CIBERFES) Instituto de Salud Carlos III Madrid Spain

## Abstract

Centenarians exhibit remarkable disease resilience despite chronic low‐grade inflammation. We investigated the inflammation‐related proteome response to acute exercise in seven centenarians (100–104 years). Exercise downregulated 52 proteins (e.g., TNF, IL10, IL1RN, CCL family members) involved in immune cell trafficking, apoptosis, and cytokine regulation. Even at the extreme end of the lifespan, humans retain molecular responsiveness to exercise, with modulation of inflammation‐related pathways.

## Graphical abstract

Targeted‐proteome analysis suggests that a single acute exercise session may attenuate inflammation at the end of the human lifespan.

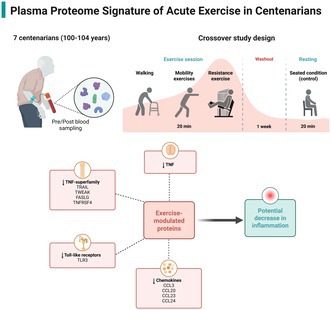

**Abbreviations**
CCL: C‐C motif chemokine ligand
FASLG: fas ligand
IL, interleukin: IL1RN, interleukin 1 receptor antagonist
TLR: toll‐like receptor
TNF: tumor necrosis factor
TNFRSF4: TNF receptor superfamily member 4
TRAIL: TNF‐related apoptosis‐inducing ligand
TWEAK: TNF‐related weak inducer of apoptosis

Centenarians offer a unique model for studying disease resilience (Olshansky et al. 2024), as they exhibit an exceptional ability to delay or escape major age‐related disorders (Summer et al. 2024; Nolen et al. 2017). A hallmark of aging is chronic low‐grade inflammation (*inflammaging*), which contributes to several conditions (López‐Otín et al. 2023). Despite higher levels of circulating inflammatory markers compared to younger adults (Basile et al. 2012; Pinti et al. 2023; Ligotti et al. 2023; Aiello et al. 2021; Zhou et al. 2022; Chulenbayeva et al. 2024), centenarians appear to avoid—or at least postpone—the detrimental effects of persistent inflammation (Ligotti et al. 2023; Aiello et al. 2021; Zhou et al. 2022).

Acute physical exercise can transiently increase inflammatory mediators, such as tumor necrosis factor (TNF) (Chow et al. 2022), followed by a compensatory anti‐inflammatory response driven in part by muscle‐released myokines such as interleukin (IL)6 (Chow et al. 2022). Thus, regular exercise (i.e., repeated acute sessions) is known to attenuate chronic inflammation (Chow et al. 2022). However, the molecular effects of exercise in centenarians remain largely unexplored. To address this gap, we examined the acute plasma proteome response to exercise in centenarians to assess whether physiological adaptability is preserved in extreme aging.

We conducted a crossover study (Figure 1A) involving seven centenarians (100–104 years, six women) from three nursing homes in Madrid, Spain. Each participant completed two sessions, separated by a one‐week washout: (1) an acute ~20‐min exercise session, including walking, mobility exercises, and high‐intensity resistance exercise (rated 8/10 on the OMNI‐resistance exercise scale); and (2) a matched‐duration rest (seated) condition (control). Blood was drawn immediately before and after each session. Plasma samples were analyzed using the Olink Explore Inflammation panel (Olink Bioscience, Uppsala, Sweden), which quantifies 368 inflammation‐related proteins.

**FIGURE 1:**
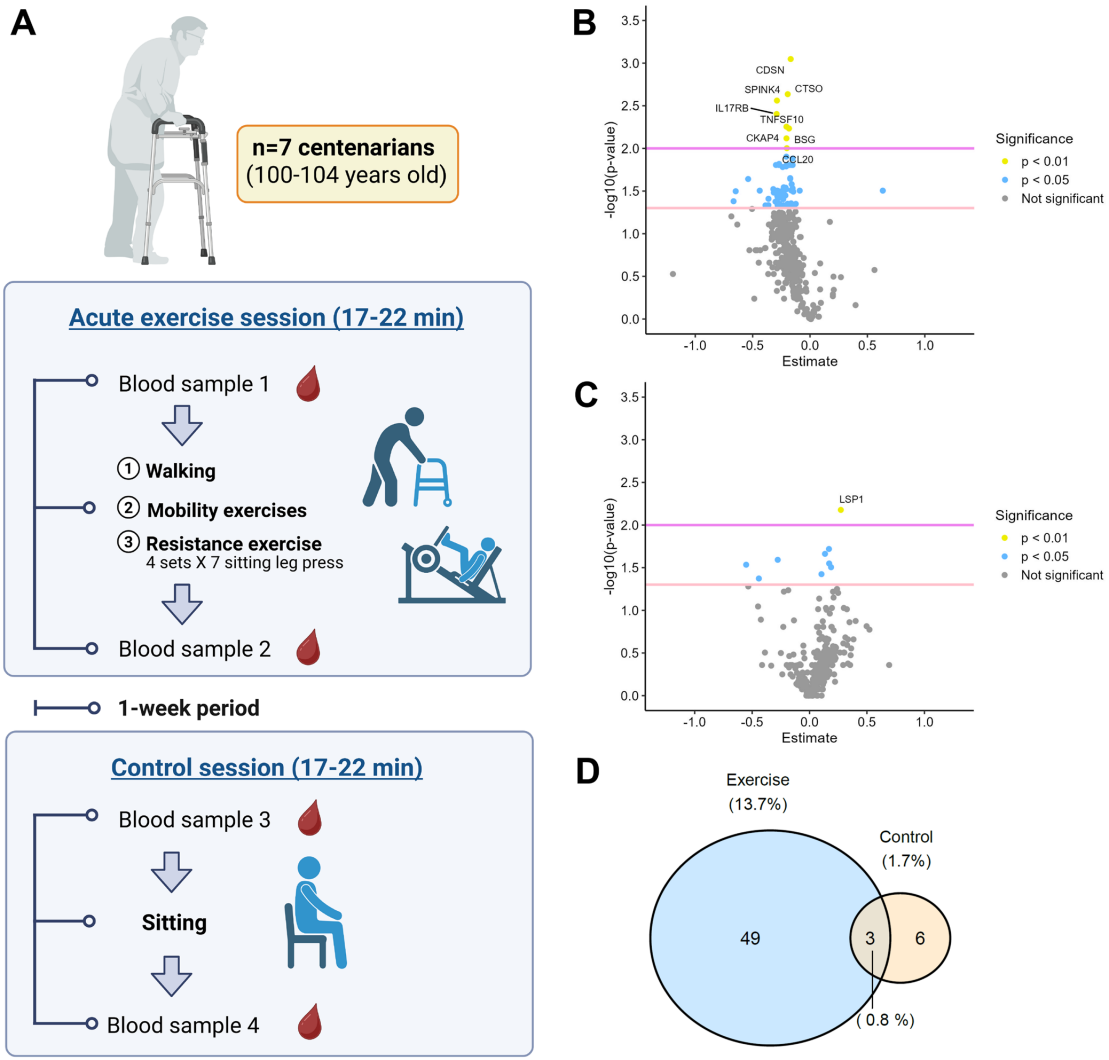
Study design and inflammation‐related proteome after acute exercise. (A) Study design. Volcano plots indicating the differentially expressed proteins after an acute bout of exercise (blood sample 1 vs. blood sample 2) (B) or a resting (control) session (blood sample 3 vs. blood sample 4) (C). Proteins with significant upregulation or downregulation are color‐coded based on thresholds for statistical significance: *p* < 0.05 (blue) and *p* < 0.01 (yellow). (D) Venn diagram displaying overlapping and differentially expressed proteins in the exercise and control sessions.

Of these, 52 proteins were significantly downregulated after exercise (Figure 1B), while only nine were altered in the control session (Figure 1C). Just three proteins overlapped between the two conditions (Figure 1D), reinforcing the specificity of the exercise response. Pathway enrichment analysis identified 10 aging‐related pathways impacted by exercise (Figure 2A), including those linked to immune cell trafficking, cytokine activity, cell death signaling, and general inflammatory regulation.

**FIGURE 2:**
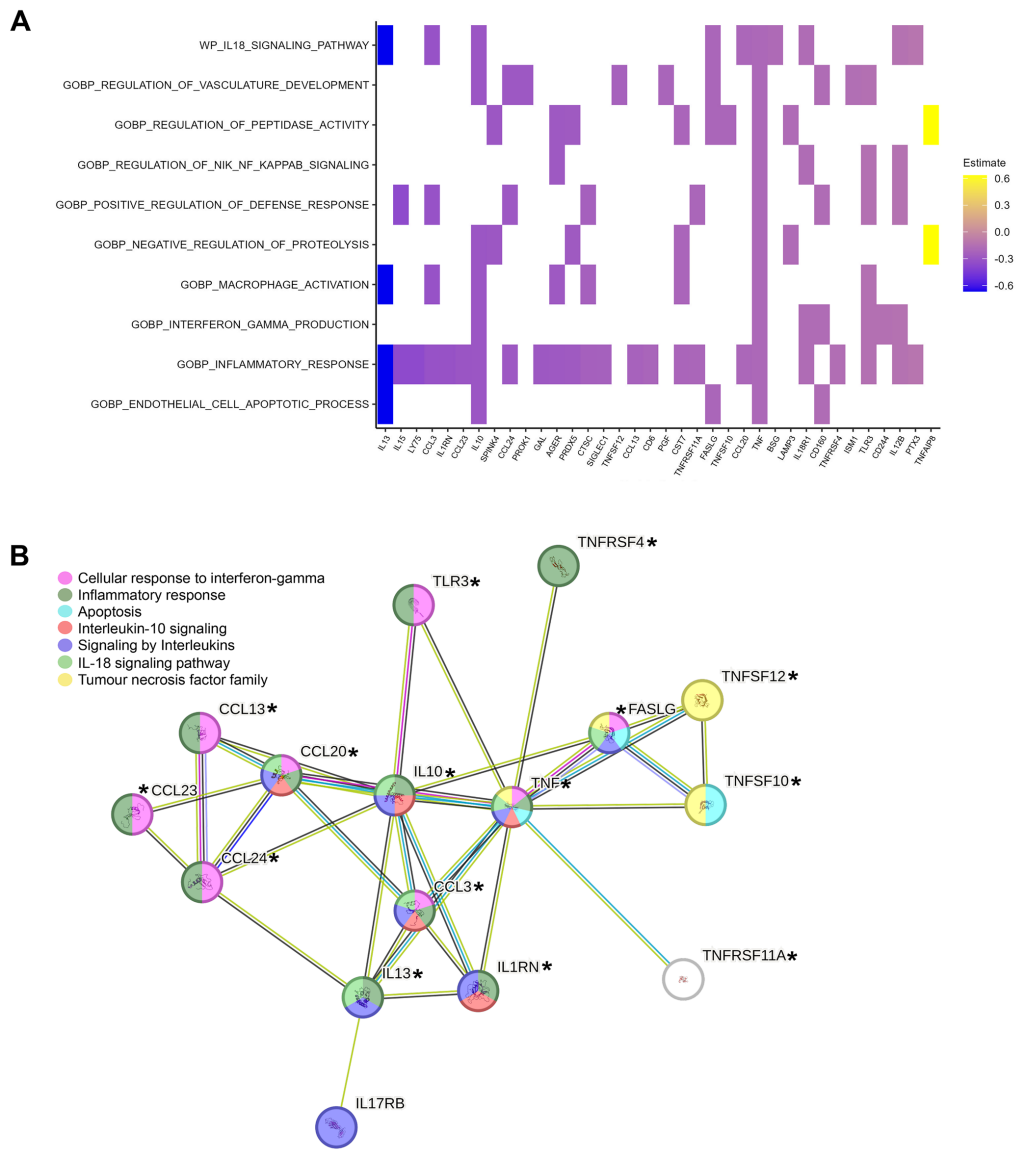
Enrichment and network analyses of inflammation‐related proteins modulated by acute exercise. (A) Heatmap of selected enriched aging‐related pathways altered by exercise. (B) Protein–protein interaction network analysis showing the 16 highly connected inflammatory proteins that were downregulated by exercise. Symbol: *indicates those proteins associated with enriched pathways (Panel A, *x*‐axis).

Protein–protein interaction network analysis revealed 16 inflammatory proteins as highly interconnected within the exercise‐modulated proteome (Figure 2B). Among them, TNF, a central immune regulator and a predictor of mortality in centenarians (Bruunsgaard et al. 2003), was significantly downregulated by exercise and emerged as the most connected node. Other significantly downregulated proteins included several members of the TNF superfamily—TNF‐related apoptosis‐inducing ligand (TRAIL, a.k.a. TNFSF10), TNF‐related weak inducer of apoptosis (TWEAK, a.k.a. TNFSF12), Fas ligand (FASLG), and TNF receptor superfamily member 4 (OX40, a.k.a. TNFRSF4)—as well as toll‐like receptor (TLR)3, all of which are implicated in immune cell apoptosis and endothelial integrity. Apoptotic pathways are essential for immune homeostasis, but their overactivation in aging is associated with immunosenescence, impaired tissue regeneration, and vascular dysfunction (Tower 2015). TNFSF10 and FASLG regulate apoptotic pathways in immune and endothelial cells, influencing lymphocyte turnover and vascular aging (Oakley and Tharakan 2014). Thus, exercise‐induced downregulation of these two proteins may indicate a transient suppression of proapoptotic signaling, potentially helping to preserve immune function and vascular stability. Additionally, TNFRSF4 is involved in T‐cell survival and sustained immune activation (Webb et al. 2016). Since persistent TNFRSF4 signaling is linked to chronic inflammation and autoimmunity (Webb et al. 2016), its suppression could contribute to immune regulation in centenarians. Moreover, the downregulation of TLR3 suggests a potential reduction in innate immune activation, given the role of this pattern recognition receptor in recognizing viral double‐stranded RNA and promoting inflammation (Sakaniwa et al. 2023). Various chemokines—for example, C‐C motif chemokine ligand 3 (CCL3), CCL20, CCL23, and CCL24—were also significantly downregulated after exercise. Considering their role in acute inflammation and immune cell chemotaxis, their suppression may reflect a transient reduction in immune cell recruitment and dampening of inflammation.

In younger individuals, an exercise session typically induces a transient inflammatory response (e.g., temporary TNF increases) followed by anti‐inflammatory effects mediated by myokines such as IL6 (Chow et al. 2022). IL6 rises with muscle contractions, leading to increased circulating IL6 levels post‐exercise (Steensberg et al. 2000). This transient increase in IL6 stimulates anti‐inflammatory cytokines, such as IL1RN and IL10 (Steensberg et al. 2003). However, in our study, plasma IL6 levels did not significantly increase following acute exercise in centenarians. This blunted response may reflect several factors including the limited ability of these individuals to exercise at sufficient intensity or duration, as well as age‐related alterations in immune signaling and myokine release—the latter possibly linked to severe sarcopenia (i.e., atrophy of skeletal muscle, the tissue where myokines are produced). Nevertheless, the decrease of inflammatory mediators, including TNF, TNFSF10, TNFSF12, FASLG, TNFRSF4, and key chemokines, suggests that acute exercise may contribute to lowering systemic inflammatory burden—which is considered a hallmark of aging resilience (López‐Otín et al. 2023). At the same time, the downregulation of some anti‐inflammatory interleukins (e.g., IL10, IL13, IL1RN) may reflect a complex regulatory effect of exercise on immune balance, influencing both pro‐ and anti‐inflammatory pathways.

The study's main limitation is the small sample size—justifiable given the rarity of centenarians and the logistical challenges of implementing exercise in these individuals. Additionally, only one postexercise blood sample was collected for ethical reasons. Albeit preliminary, our results suggest that humans retain molecular responsiveness to exercise even at extreme ages. Notably, three centenarians died within 3–10 months after the study, emphasizing the importance of further research into the role of exercise in late‐life resilience.

In an era where regenerative medicine and costly interventions aim to extend lifespan beyond known limits, our findings highlight the potential of exercise to elicit meaningful molecular responses, even at the very end of life, possibly modulating inflammatory pathways. To expand on these findings, extended post‐exercise sampling and further longitudinal studies are warranted to explore whether repeated exercise sessions can sustain or enhance these effects. Future studies incorporating immunophenotyping, myokine profiling, and functional immune assays could provide deeper insights into immune adaptation and resilience mechanisms in exceptional aging.

## 1 Experimental Procedures

Detail on exercise protocol, proteomic analysis, and statistical methods are available in Supporting Information. Supplementary Files include: (1) online methods (Data S1), (2) video of the exercise protocol (Data S2), (3) raw dataset (Data S3), (4) statistical results for all 358 proteins (Data S4), and (5) pathway enrichment analysis results from the exercise condition (Data S5).

## Author Contributions

Alejandro Lucia and Carmen Fiuza‐Luces conceived the project. Alejandro Lucia, Lidia Brea‐Alejo, and Carmen Fiuza‐Luces designed the experiments. Lidia B. Alejo, María Rosado Muñoz, Itziar Pagola Aldazabal, David Barranco‐Gil, Gabriel Rodríguez‐Romo, Natalia Yanguas‐Casás, Alejandro Lucia, and Carmen Fiuza‐Luces performed data collection. Abel Plaza‐Florido, Alejandro Santos‐Lozano, and Inmaculada Pérez‐Prieto analyzed the experiments. Abel Plaza‐Florido, Pedro Carrera‐Bastos, and Alejandro Lucia wrote the manuscript. All authors commented and approved the manuscript.

## Conflicts of Interest

The authors declare no conflicts of interest.

## Supporting information


**Data S1.** Online methods.

**Data S2.** Video demonstrating the exercise protocol.

**Data S3.** Raw dataset used for analysis.

**Data S4.** Statistical results of all 358 proteins included in the study.

**Data S5.** Pathway enrichment analysis results from the exercise condition.

## Data Availability

All data supporting the findings of this study are available in the article and Supporting Information.

## References

[acel70152-bib-0001] Aiello, A. , G. Accardi , S. Aprile , et al. 2021. “Pro‐Inflammatory Status Is Not a Limit for Longevity: Case Report of a Sicilian Centenarian.” Aging Clinical and Experimental Research 33: 1403–1407. 32577916 10.1007/s40520-020-01628-7

[acel70152-bib-0002] Basile, G. , I. Paffumi , A. G. D'Angelo , et al. 2012. “Healthy Centenarians Show High Levels of Circulating Interleukin‐22 (IL‐22).” Archives of Gerontology and Geriatrics 54: 459–461. 21640395 10.1016/j.archger.2011.05.004

[acel70152-bib-0003] Bruunsgaard, H. , K. Andersen‐Ranberg , J. v. B. Hjelmborg , B. K. Pedersen , and B. Jeune . 2003. “Elevated Levels of Tumor Necrosis Factor Alpha and Mortality in Centenarians.” American Journal of Medicine 115: 278–283. 12967692 10.1016/s0002-9343(03)00329-2

[acel70152-bib-0004] Chow, L. S. , R. E. Gerszten , J. M. Taylor , et al. 2022. “Exerkines in Health, Resilience and Disease.” Nature Reviews. Endocrinology 18: 273–289. 10.1038/s41574-022-00641-2 PMC9554896 35304603

[acel70152-bib-0005] Chulenbayeva, L. , Y. Ganzhula , S. Kozhakhmetov , et al. 2024. “The Trajectory of Successful Aging: Insights From Metagenome and Cytokine Profiling.” Gerontology 70: 390–407. 38246133 10.1159/000536082 PMC11008724

[acel70152-bib-0006] Ligotti, M. E. , G. Accardi , A. Aiello , et al. 2023. “Sicilian Semi‐ and Supercentenarians: Identification of Age‐Related T‐Cell Immunophenotype to Define Longevity Trait.” Clinical and Experimental Immunology 214: 61–78. 37395602 10.1093/cei/uxad074 PMC10711357

[acel70152-bib-0007] López‐Otín, C. , M. A. Blasco , L. Partridge , M. Serrano , and G. Kroemer . 2023. “Hallmarks of Aging: An Expanding Universe.” Cell 186: 243–278. 36599349 10.1016/j.cell.2022.11.001

[acel70152-bib-0008] Nolen, S. C. , M. A. Evans , A. Fischer , M. M. Corrada , C. H. Kawas , and D. A. Bota . 2017. “Cancer‐Incidence, Prevalence and Mortality in the Oldest‐Old. A Comprehensive Review.” Mechanisms of Ageing and Development 164: 113–126. 28502820 10.1016/j.mad.2017.05.002 PMC7788911

[acel70152-bib-0009] Oakley, R. , and B. Tharakan . 2014. “Vascular Hyperpermeability and Aging.” Aging and Disease 5: 114–125. 24729937 10.14336/AD.2014.0500114 PMC3966670

[acel70152-bib-0010] Olshansky, S. J. , B. J. Willcox , L. Demetrius , and H. Beltrán‐Sánchez . 2024. “Implausibility of Radical Life Extension in Humans in the Twenty‐First Century.” Nature Aging 4: 1635–1642. 39375565 10.1038/s43587-024-00702-3 PMC11564081

[acel70152-bib-0011] Pinti, M. , L. Gibellini , D. Lo Tartaro , et al. 2023. “A Comprehensive Analysis of Cytokine Network in Centenarians.” International Journal of Molecular Sciences 24: 2719. 36769039 10.3390/ijms24032719 PMC9916918

[acel70152-bib-0012] Sakaniwa, K. , A. Fujimura , T. Shibata , et al. 2023. “TLR3 Forms a Laterally Aligned Multimeric Complex Along Double‐Stranded RNA for Efficient Signal Transduction.” Nature Communications 14: 164. 10.1038/s41467-023-35844-2 PMC9834221 36631495

[acel70152-bib-0013] Steensberg, A. , C. P. Fischer , C. Keller , K. Møller , and B. K. Pedersen . 2003. “IL‐6 Enhances Plasma IL‐1ra, IL‐10, and Cortisol in Humans.” American Journal of Physiology. Endocrinology and Metabolism 285: E433–E437. 12857678 10.1152/ajpendo.00074.2003

[acel70152-bib-0014] Steensberg, A. , G. van Hall , T. Osada , M. Sacchetti , B. Saltin , and B. Klarlund Pedersen . 2000. “Production of Interleukin‐6 in Contracting Human Skeletal Muscles Can Account for the Exercise‐Induced Increase in Plasma Interleukin‐6.” Journal of Physiology 529, no. Pt 1: 237–242. 11080265 10.1111/j.1469-7793.2000.00237.x PMC2270169

[acel70152-bib-0015] Summer, S. , M. Borrell‐Pages , R.‐M. Bruno , et al. 2024. “Centenarians‐The Way to Healthy Vascular Ageing and Longevity: A Review From VascAgeNet.” Geroscience 47: 685–702. 39725804 10.1007/s11357-024-01467-8 PMC11872877

[acel70152-bib-0016] Tower, J. 2015. “Programmed Cell Death in Aging.” Ageing Research Reviews 23: 90–100. 25862945 10.1016/j.arr.2015.04.002 PMC4480161

[acel70152-bib-0017] Webb, G. J. , G. M. Hirschfield , and P. J. L. Lane . 2016. “OX40, OX40L and Autoimmunity: A Comprehensive Review.” Clinical Reviews in Allergy and Immunology 50: 312–332. 26215166 10.1007/s12016-015-8498-3

[acel70152-bib-0018] Zhou, L. , M. Ge , Y. Zhang , et al. 2022. “Centenarians Alleviate Inflammaging by Changing the Ratio and Secretory Phenotypes of T Helper 17 and Regulatory T Cells.” Frontiers in Pharmacology 13: 877709. 35721185 10.3389/fphar.2022.877709 PMC9203077

