# Supplementary material for: Even at 100+: Acute Exercise Modulates Inflammatory Pathways in Centenarians
Source: Aging Cell. 2025 Jul 15;24(9):e70152. doi: 10.1111/acel.70152 (PMC12419837; doi:10.1111/acel.70152)
Supplement: Supplementary file 1 — Data S1. Online methods. [file ACEL-24-e70152-s005.zip › acel70152-sup-0001-DataS1/Supplementary File 1_Online methods_centenarians_24_Jun_2025.docx]

**Online Supporting Information**

***Study Design***

This crossover study included seven centenarians (100-104 years, six women) from three nursing homes in Madrid, Spain. Two experimental conditions were tested, separated by a one-week ‘washout’ period:

1. Acute exercise session: Participants completed in the gymnasium of the relevant nursing home, a 20-minute session (range, 17-22 minutes) consisting of walking, mobility exercises, and four sets of seven repetitions of high-intensity resistance exercise (sitting leg press), corresponding to a score of 8 (‘hard’) on the 0-10 OMNI-resistance exercise scale for perceived exertion.
2. Control session: Participants remained seated quietly for the same duration (~20 minutes) as the exercise session, performed in the same location and at the same time of day.

Both sessions were conducted in the morning (between ~10:00 am and 12:00 pm) to control for diurnal variation. Blood samples were collected immediately before and after each session.

Given the high vulnerability of this population, implementing the exercise protocol required substantial logistical support, including individualized supervision, adaptive safety strategies, and continuous monitoring to ensure participant well-being. A video of the exercise protocol is available at:

<https://drive.google.com/file/d/18CmuF2jgHHYoB6wNJDQGc51OgA1ZEDHL/view?usp=sharing>

*Blood Sample Collection and Processing*

Blood was collected in EDTA tubes by venipuncture and immediately centrifuged at 1000 g for 10 minutes at 4°C. Plasma was aliquoted and stored at −80°C until analysis. One microliter of plasma per participant was used for proteomic analysis. All samples were processed under the same conditions to minimize technical variability.

*Targeted Proteomic Analysis*

Plasma proteomic profiling was performed using the Olink® Explore Inflammation panel (Olink Bioscience, Uppsala, Sweden), which quantifies 368 inflammation-related proteins using Proximity Extension Assay (PEA) technology (<https://olink.com/products/olink-explore-3072-384>). This highly sensitive and specific method uses pairs of antibodies labelled with DNA oligonucleotides that bind target proteins. When in proximity, the oligonucleotides hybridize and are extended by DNA polymerase to generate unique DNA sequences, which are then amplified and quantified via microfluidic quantitative polymerase chain reaction (PCR).

Of the 368 proteins measured, 10 (BCL2L11, BID, PTPRM, CD40LG, LTA, IDS, RAB6A, CLEC7A, HGF, MGLL) had zero counts across all samples and were excluded. Thus, 358 proteins were included in the statistical analysis. Normalized Protein Expression (NPX) values—Olink’s arbitrary unit presented on a log_2_ scale—were used. NPX values are directly proportional to protein concentration, with a one-unit NPX difference reflecting a twofold change. Details about the PEA technology (including assay performance, intra- and inter-assay coefficients of variation, and detection limits) are available on the manufacturer’s website (<https://www.olink.com/>).

Note: Throughout this article, we use HGNC (HUGO Gene Nomenclature Committee) gene symbols (e.g., TNFSF10, IL10, IL6, IL1RN, TNFRSF4) to refer to proteins, not genes. Although these are technically gene identifiers, they are widely adopted in proteomics and bioinformatics platforms (e.g., Olink, STRING, MSigDB) as standard shorthand for the corresponding protein products. To reflect this usage, we present them in non-italicized uppercase format when denoting proteins.

*Statistical Analysis*

All analyses were performed using R (v.4.2.1). One sample did not meet quality control standards and was excluded. The OlinkAnalyze R package was used to identify differentially expressed proteins (DEPs) before and after the acute intervention in both the exercise and control groups. Normality was tested using the Shapiro-Wilk test. For normally distributed proteins, paired t-tests were applied (olink_ttest). For non-normal distributions, the Wilcoxon signed-rank test was used (olink_wilcox). A *p*-value < 0.05 was considered statistically significant.

Pathway enrichment analysis was conducted using Over-Representation Analysis (ORA) through MSigDB, utilizing the olink_pathway_enrichment function and the clusterProfiler package from Bioconductor. Gene sets were derived from Gene Ontology (GO), Kyoto Encyclopedia of Genes and Genomes (KEGG), and Reactome databases. This approach allowed identification of biological processes and pathways significantly impacted by exercise-induced protein modulation.

The protein-protein interaction (PPI) network was constructed and visualized using STRING (v.12.0) to assess the connectivity and potential functional relationships among DEPs identified in the exercise condition.

Data visualization included volcano plots displaying the statistical significance and magnitude of protein expression changes, heatmaps of enriched pathways (generated using the olink_pathway_heatmap function from the OlinkAnalyze package and further customized with ggplot2), and Venn diagrams illustrating overlapping DEPs between conditions (created using the VennDiagram package).

*Ethical Considerations*

The study protocol was approved by the relevant ethics committee (reference number A06/23). All participants provided written informed consent. The study complied with the principles of the Declaration of Helsinki.
